# Meritocracy, Recognition and Double Consciousness: Why Black and Muslim Italians Move to (and Sometimes Leave) Post‐Brexit Britain

**DOI:** 10.1111/1468-4446.70064

**Published:** 2025-11-12

**Authors:** Simone Varriale, Michela Franceschelli

**Affiliations:** ^1^ Dept. of Criminology Sociology and Social Policy SSSH Loughborough University Loughborough UK; ^2^ UCL Social Research Institute Thomas Coram Research Unit University College London London UK

**Keywords:** Black and Muslim Italians, double consciousness, ideology, meritocracy, racism, recognition

## Abstract

This article rethinks meritocratic ideology as practical knowledge that transforms through biographies of social and geographical mobility. Drawing on 37 interviews with Black and Muslim Italians living in Britain or returned to Italy, the article shows that meritocracy is rarely invoked as a coherent ideology but works as practical, embodied commonsense about the world order, with Britain leading a hierarchy of European societies. The article explores three dimensions of meritocratic commonsense and racialised minorities' double‐consciousness (Du Bois). First, 'meritocratic Britain' is not simply a neoliberal narrative, but draws from postcolonial, intergenerational histories of family migration that include desires for equality and security. Second, participants' encounters with British racism do not necessarily challenge beliefs in meritocratic Britain, as being racialised as 'foreigners' in Italy leaves deeper scars on their sense of identity, belonging and recognition. Third, meritocratic Britain can lose emotional resonance when participants feel desires for connectedness and home that are not satisfied by occupational and educational mobility. By centring racialised minorities' double‐consciousness, practical knowledge and struggles for recognition, the article highlights the limitations of false consciousness, misinformation and psychological compensation as explanations for meritocratic belief. Moreover, it unravels how meritocratic narratives transform across life stages.

## 1 Introduction

Sociology and related social sciences are increasingly concerned with a paradox: belief in meritocracy ‐ the notion that economic rewards reflect individual effort and talent ‐ appears to strengthen as inequality rises, particularly in highly unequal societies like the US and UK (Littler 2017; Mijs 2021). Quantitative studies suggest that many continue to hold positive views about the role of ‘hard work' in shaping life chances, which underscores the ideological force of meritocracy. Yet, these conclusions remain contested, given the limits of survey‐based attitudinal measures (Bartram 2023; Zhu 2024).

This article approaches this puzzle differently. Drawing on interviews with Black and Muslim Italians living in post‐Brexit Britain (or who returned to Italy), we rethink meritocracy as a *practical, embodied commonsense* that is reshaped through experiences of social and geographical mobility. Elaborating on Du Bois's notion of double‐consciousness (1903/2007), we show how self‐formation in structurally racist societies sustains emotional investment in meritocratic narratives, as racialised minorities see ‘hard work' as a path to social respect and *recognition* (Fraser and Honneth 2003). Yet, this investment co‐exists with nuanced understanding of racial inequalities, while belief in meritocracy proves to be context‐dependent, relational and temporally changing.

Our findings highlight three dynamics. First, post‐1970s neoliberalism offers a historically limited frame for understanding our participants' relationship with ideas of merit. These draw on longer‐term, intergenerational histories of migration, premised on aspirations towards equality and security. Second, participants' encounters with British racism do not necessarily undermine belief in meritocracy, as the experience of being racialised as *stranieri* (foreigners) in Italy leaves deeper wounds on their sense of self, leading to a search for recognition abroad. Third, meritocratic narratives can be challenged by desires for connectedness, belonging and home, which are not satisfied solely by occupational and educational mobility (the practices historically tied to meritocratic recognition; Littler 2017).

Methodologically, the article focuses on how meritocratic ideology shapes a tangible social practice: international migration. Youth migration is tied to aspirations of self‐improvement and upward mobility (Varriale 2023; Franceschelli 2022). Thus, it offers a more defined context (compared to attitudinal survey questions) for exploring the power of meritocracy across temporal, geographical and social dimensions. By focusing on Black and Muslim Italians, the article also addresses a group of minoritised Europeans who remain at the periphery of debates on racism and inequality (Hawthorne 2022), and whose views about Italy, Britain and meritocracy are influenced by experiences of racism that cross borders and generations.

Rather than approaching meritocracy as a problem of (mis)information, false consciousness or psychological compensation ‐ the main explanations provided in the literature ‐ we provide a less reductionist account centred on practical knowledge, struggles for recognition and biographical change. The next section situates this study within scholarship on meritocracy and inequality. We then discuss Du Bois's relevance for our theoretical framing, the context of our study (migration and racism across Italy and Britain), and the article's methodology, before turning to findings and conclusions.

## 2 Beyond False Consciousness: Meritocracy as Practical Knowledge

Discussions of meritocracy sit at the crossroad between different fields, with significant disconnection between survey‐based studies of meritocratic beliefs and qualitative work on meritocratic narratives. This section discusses the tensions between these literature, before advocating for a focus on meritocracy as practical and embodied commonsense.

Survey studies explore the power of meritocratic ideology especially via questions about ‘hard work' and its importance for individual success (Mijs 2021; Morris et al. 2022; but see Bartram 2023). This literature explains meritocracy's power via explicit or implicit notions of false consciousness, broadly defined as ‘beliefs which are false but make sense of the world [individuals] inhabit' (Mijs and Usmani 2024, 58). Research on the upper‐middle classes shows that they report stronger belief in hard work compared to more disadvantaged groups (Bucca 2016; Bottero 2019, 25–53). The working classes, however, are also invested in meritocracy, which has been explained as a form of psychological compensation: meritocracy provides a way of rationalising one's disadvantage as deserved (Solt et al. 2016, 2) or to protect one's self‐esteem by ‘hold[ing] out the promise of future advancement' (Morris et al. 2022, 423). Either way, meritocracy remains conceptualised as a false consciousness that distorts the ‘real' causes of inequality. Mijs and Usmani (2024) recently elaborated on meritocracy as a (mis)information problem. They argued that people underestimate causes and levels of inequality because they are embedded in socially and geographically distant networks. Belief in meritocracy is thus an ‘inferential error' sustained by lack of contact across socio‐economic divisions.

As argued by Fercovic (2022: 119), survey‐based literature on meritocracy risks ‘neglecting the *contextual specificities* and the *meaning‐making processes* in which these phenomena are embedded [original emphasis]'. By contrast, work inspired by Gramsci's hegemony (1975) and Hall (1986) highlights the importance of a more contextually sensitive take on ideology. These studies agree that meritocracy contributes to the legitimation of growing inequalities (Meghji and Saini 2018), but unlike stronger notions of false consciousness, they conceptualise hegemony as fuzzy, potentially relying on contradictory ideas and open to transformation (Hall and O'Shea 2013). Hegemony is also a composite product of different historical narratives. Indeed, while ideas of self‐responsibility and individual effort were key to the legitimation of post‐1970s neoliberalism, meritocracy also draws on earlier social‐democratic discourses, which saw state infrastructures as means to guarantee equality of opportunity (Littler 2017, 51–53); a connection this article will unpack further.

The notion of hegemony resonates with recent qualitative literature, which shows that individuals can mobilise both meritocratic and structuralist narratives of (in)equality in different contexts (Andersen et al. 2021; Asahina 2021). Lamont and et al. (2016) show that meritocratic narratives of self‐resilience and self‐improvement co‐exist with critique of racial inequalities among Black Americans. Fercovic's work on Chile (2022) shows that upwardly mobile working‐class individuals do not misjudge how inequality affects their lives, but forge moral stories about their families as ‘people of effort', rethinking merit as a combination of individual work, family support and luck. Similarly, Ho (2024) approaches meritocracy as a cultural narrative that allows working‐class youth to construct ‘agentic selves'. Her participants are aware of being structurally disadvantaged, but mobilise ‘hard work' as a pragmatic strategy to keep going in life.

Expanding on this literature and our previous research (Varriale 2023; Franceschelli 2024), we address meritocratic ideology as part of *practical commonsense* that develops via socialisation in national, classed and racialised contexts, including family histories of migration (see empirical sections). Practical logic operates via ‘often imprecise but systematic principle[s] of selection' (Bourdieu 1990, 102). These principles of ‘vision' of the social world are embodied in the *habitus*, a ‘system of structured, structuring dispositions […] which is constituted in practice and is always oriented towards practical functions' Bourdieu (1990, 52).

Meritocratic beliefs can thus be conceptualised as cognitive‐emotional 'maps' shaped by trajectories of social and geographical mobility. Drawing on Bourdieu's emphasis on the embodied, pre‐reflexive and fuzzy nature of practical logic, we demonstrate how participants' imaginaries of meritocratic Britain gained significance through the experience of growing up as *racialised* Italians. These imaginaries then evolved as participants navigated migration and the realities of British society, within new *fields of practice* (Bourdieu 1990).

An emphasis on practical knowledge also helps unpack the contradictory, ‘intertwined' character of cultural narratives (Lamont and et al. 2016, 82). As this article shows, in the context of migration, meritocracy is not mentioned as a coherent ideology, but as part of wider stories about the postcolonial world order, which associate the Global North with more economically advanced, meritocratic societies, and Southern geo‐political regions (including Southern Europe) as 'backwards'. These understandings of world hierarchies have been influential in post‐2008 Italy (Varriale 2023), but are documented also in scholarship on the historical framing of global inequalities in Europe (Boatcă 2015).

By examining the *tensions* between practical knowledge and lived experience, and how they unfold across biographical trajectories, this article explores ideology's power in more dynamic ways than survey‐based literature. Additionally, it moves beyond the focus of some qualitative studies on the alignment between ideology and experience; namely, when individuals invoke meritocratic narratives as explanations for (achieved) upward mobility (Fercovic 2022; Meghji and Saini 2018).

## 3 Racism, Double Consciousness and Meritocratic Recognition

The literature discussed above largely ignores the linkages between meritocratic ideology and questions of recognition and racism, despite a history of ‘vernacular' forms of self‐resilience within the Black Diaspora (Gilroy 2013). Yet this neglected history might help studies of meritocracy better understand the *limitations* of ideological power and how minoritised communities adapt hegemonic narratives to their search for recognition and equality.

Du Bois's work (1903/2007) provides an important link between recognition^1^ and *racialised self‐formation* (Itzigsohn and Brown 2015; Meer and Du Bois 2019). He famously began his reflection on ‘double‐consciousness' by discussing how Black Americans develop unique insights into the workings of racism:

It is a peculiar sensation, this double‐consciousness, this sense of always looking at one's self through the eyes of others, of measuring one's soul by the tape of a world that looks on in amused contempt and pity. One ever feels his twoness—an American, a Negro; two souls, two thoughts, two unreconciled strivings; two warring ideals in one dark body, whose dogged strength alone keeps it from being torn asunder.

(Du Bois, 1903/2007: 8)

Du Bois's emphasis on racialised minorities' 'dogged strength' is especially relevant for debates about meritocratic ideology. His analysis does not stop at self‐stigmatisation, but dialectically accounts for the development of a 'second sight' into the phenomenology of racism. Misrecognition is thus not passively accepted but actively struggled with, and potentially challenged (Meer and Du Bois 2019). This resonates with findings on Black Americans' ‘dual perspective' regarding both the existence of racial inequalities and the value of hard work (Lamont and et al. 2016). It also aligns with evidence of growing activism among minoritised Europeans, as the ‘unreconciled strivings' of feeling Black, Muslim *and* Italian are increasingly documented (Hawthorne 2022; Pesarini 2021). As discussed below, the experience of being misrecognised as 'foreigners' in Italy makes narratives of meritocratic Britain appealing to participants, but only under certain structural, relational and temporal conditions.

A Du Boisian perspective on recognition thus challenges discussions of meritocracy as a defence mechanism among the less privileged, which stop at self‐stigmatisation and system justification (Jost 2019). This does not account for ambivalence, critique or resistance, while theoretically more sophisticated accounts of meritocracy as a cultural script (Ho 2024) do not address the specificity of *racialised self‐formation* and its consequences across the lifecourse. The concept of double consciousness can thus support an analysis of meritocracy's power which acknowledges the enduring impact of racism, while remaining attentive to questions of agency, biographical change and social‐geographical mobility.

## 4 Context: From Colour‐Blind Italy to Multicultural Britain

Migrations between Italy and Britain are relevant to the meritocracy debate because they highlight the possibility, so far neglected, that people's ‘sense of inequality' (Bottero 2019) is not fully constrained by national borders. Indeed, after the 2008 economic crisis, wage stagnation, labour market precarisation and growing unemployment fostered new emigrations from Italy to Britain; a destination that Italian media frequently associated with better opportunities and a more meritocratic culture (Varriale 2023). While Italy's structural problems remain ongoing, Britain experienced major economic and political crises since Brexit. This allows us to explore the tensions between meritocratic ideology and lived experience across different socio‐political contexts.

Both Italy and Britain have colonial histories that inform their ethno‐racial stratification. However, institutional and everyday racism manifest differently in these contexts. Britain has a history of postcolonial migrations dating to the 1950s: racial equality legislation was introduced from the mid‐1960s and data collection about ethno‐racial inequalities has become institutionalised (Finney and et al. 2023; Mirza and Warwick 2024, 428–430). By contrast, Italian institutions do not collect data on ethno‐racial inequalities, while the word ‘race' (*razza*) and Italy's colonial history have been erased from public debate throughout the post‐war period (Pesarini 2021). Moreover, large‐scale immigration from the 1980s was met with increasingly restrictive citizenship and immigration policies (Lombardi‐Diop and Romeo 2015; Obasuyi 2025). Compared to Britain, Italy thus resembles wider patterns of European colour‐blindness (Beaman 2017; Balogun 2023). This can normalise overt everyday racism (Benson and Lewis 2019) and policies with structurally racist impacts. Indeed, Black and minoritised Italian activists have increasingly criticised the contradiction between colour‐blind institutional practices and everyday racist violence (Ghebremariam Tesfau' and Picker 2021; Obasuyi 2025).

In Britain, despite persisting ethno‐racial inequalities (especially for Black Caribbean, Black African, Bangladeshi and Pakistani minorities), growing educational mobility sustained the emergence of a Black and South Asian middle class in the last 25 years (Meghji and Saini 2018). A similar process was not visible in Italy during the 2010s, when participants moved abroad (see Asma's discussion below). Moreover, racialised minorities remain concentrated in lower‐status manual and care work in Italy (Obasuyi 2025). This was a common experience among participants' parents, which fed into worries about their children's future.

These historical trends sustained narratives that associate Britain with more opportunities and meritocracy (as for white Italians). However, for Black and minoritised Italians these narratives include expectations of more institutional support towards equality and multiculturalism, and more respectful everyday interactions. These narratives circulated within participants' transnational family networks while growing up in Italy (e.g., through visits to family members in Northern Europe). Yet they became more salient during the 2010s, when Italy's worsening economy fostered new migrations (Varriale 2023; Franceschelli 2024).

## 5 Methodology

This article draws on 37 biographical interviews with Black, Muslim and other minority ethnic Italians who moved to Britain since the 2008 economic crisis. Sampling followed a theoretical rationale: we recruited participants exposed to different forms of racialisation in Italy (Morning and Maneri 2022). We used the concept of ‘second generation Italians' in recruitment materials, as this concept is significantly established in Italian public discourse on racialised minorities and, in previous research, facilitated recruitment of participants with different ethno‐national and religious backgrounds (Varriale 2025). Furthermore, the principle of *jus sanguinis* in Italian citizenship legislation prevents all children of migrants from becoming legally Italian until 18 (or until a parent becomes Italian), leading to shared experiences of *legal violence* (Menjívar and Abrego 2012) across ethno‐racial backgrounds, as discussed later.

The sample includes participants with West African (13), East African (3), North African (5), South Asian (8), Latin American (3), Middle Eastern (1) and Eastern European (4) backgrounds. Women are overrepresented (25) and only one participant identified as non‐binary. Most participants had lived in Britain for 3–10 years when interviewed and moved abroad during their twenties or thirties; 8 participants moved abroad with their families before their twenties. Six participants had left Britain when interviewed, but the topic of ‘leaving' was also discussed by 6 participants living in Britain.

The majority of participants studied or were studying at university level (27). However, their families worked predominantly in working‐class sectors in Italy. The majority had factory, cleaner or care jobs, while a minority (9) were or had been self‐employed (mostly as small business owners or skilled workers). Only 3 participants with mixed backgrounds had (white) fathers in white collar jobs. This is consistent with research on immigration and stratification in Italy: both non‐EU and Eastern European migrants are more likely to be in working‐class and precarious occupations (Panichella et al. 2023; Obasuyi 2025).

Participants were contacted through social media, via public recruitment or individual messages. This strategy expanded the project's geographical focus, an important step given the paucity of official data on racialised Italians in Britain. Only 11 participants lived or had lived in London; the majority lived around areas with established minoritised communities (e.g., Manchester, Leicester and Birmingham). Since Brexit, all participants except one had obtained pre‐settled or settled‐status. This article does not address the complexities of pre/settled status (Zambelli et al. 2023), but it means that participants felt they had a secure legal status when interviewed.

The first author conducted online interviews via Teams between late 2023 and mid‐2024, each lasting an average of 80 min. Following a strategy developed in previous research (Varriale 2025), we asked general questions about motivations for emigration, life in Britain and plans for the future, and more specific questions about educational and professional trajectories (before and after migration), relationships in Britain and Italy, and experiences of Brexit and cost of living. We asked a question about reasons for leaving Britain when relevant, then reconstructed participants' trajectories across national contexts.

We did not ask questions about meritocracy. Following previous work (Varriale 2023), we focussed on how participants spontaneously invoked meritocratic commonsense within broader narratives and in relation to specific social fields and lifecourse transitions. This strategy has the advantage of tapping into practical sense's fuzziness and contextual dimension. Moreover, the interview's biographical design addresses issues of temporality and change, which, as discussed above, remain underresearched in debates on meritocracy.

To prevent harm, we avoided asking questions about personal experiences of racism. However, Black, Muslim and non‐white^2^ participants frequently addressed the connections between emigration and being treated like *stranieri* (foreigners) in Italy. The project was presented as aiming to document the ‘diversity' of Italian emigration, which potentially suggested sympathetic listeners. The interviewer and first author is a white, male Italian migrant with a Southern Italian, low‐middle class background. While he felt that sharing language, cultural references and experiences of migration to Britain facilitated discussion, whiteness^3^ also affected discussion in significant ways. As discussed below, some participants suggested that the experience of growing up as 'foreigners' in Italy needed further explanation, given that the interviewer did not share it. This potentially produced richer narratives about the experiences that are central to this article. However, participants were also relatively young (between their twenties and early forties) and were striving to achieve upward mobility. This might have limited their willingness to disclose more difficult experiences in Britain, especially to a white co‐national with a middle‐class job. Our sampling strategy might also sustain self‐selection of participants with ‘positive' experiences of migration. Yet, ambivalences and critiques about Britain emerged nonetheless, while returnees were even more explicit about what changed earlier perceptions of Britain's meritocracy.

Interviews were analysed inductively: the links between meritocratic Britain, recognition and racism progressively emerged through repeated rounds of reading and analysis across the sample. The project received ethical approval before fieldwork started. All names used are pseudonyms; some identifying details were changed to protect participants' identity. All interviews except two were conducted in Italian; the first author translated excerpts for this article.

## 6 Before Neoliberalism: Postcolonial Narratives of Meritocratic Britain

As anticipated above, rather than discussing meritocracy as an abstract principle, participants drew on meritocratic commonsense by referencing broader cultural scripts (Lamont and et al. 2016) about modern multicultural societies, which allegedly facilitate upward mobility for racialised minorities. This section shows that this narrative is not simply a product of late‐1970s neoliberalism, but draws on earlier intergenerational stories of migration towards Western Europe, which accommodate social‐democratic notions of equality and institutional support.

Asma lived in various parts of Britain during the past 10 years. When explaining why she left Italy, she signalled the power of London in the story of meritocratic Britain, but clarified that she sees ‘meritocracy' as a more general quality of ‘the UK':

I liked the fact that it's a truly multicultural city, I really met people from all over the world, and then I liked the aspect of meritocracy, which I believe exists throughout the UK. Unfortunately it's something I haven't found in Italy […] you can see it just by looking at the jobs that foreigners have, it's very difficult to find, for example, a Moroccan working as a lawyer in Italy. Maybe it's also because immigration is a more recent phenomenon in Italy? There are many more generations in the UK, which definitely has a big impact, but the first thing I noticed is that in England foreigners are much more integrated and hold very important jobs.

Asma's discussion of Britain's ‘meritocracy' draws from practical knowledge that is common also among white Italian migrants (Varriale 2023; Franceschelli 2024). However, as an Italian with Moroccan (Muslim) parents, Asma approaches the ‘Italian' story about meritocratic Britain from a racialised position. Britain is thus also a ‘multicultural' country where, contrary to Italy, one can see a ‘Moroccan lawyer' and other 'foreigners' in ‘very important jobs', namely, a country where upward mobility for racialised minorities is easier.

Although research on meritocratic ideology connects it to post‐1970s neoliberalism, participants drew on a more composite, intergenerational narrative with postcolonial and post‐war undertones. Asma alludes to the several 'generations' of multicultural Britain (vs. Italy, a country that experienced growing immigration only from the 1980s). However, stories about the 'opportunities' offered by the former colonial metropole already circulated among participants' families and in transnational family networks. Giovanni mentioned how his dad, before leaving Ghana, already 'planned to not stay too long in Italy and go to England':

My father saved a lot of money, he came to Italy, started working, then you know how it is, you have certain dreams, then we [children] came along, so they weren't ready until we grew up. Then they thought about providing better educational opportunities for [their] children, right? It was time to send them to England […] my father decided that it was the best thing to do at that time, like many people maybe he saw how the economic situation in Italy was declining.

Giovanni situates his father's 'dreams' of opportunity and educational mobility, particularly for his children, in a longer historical perspective than the one acknowledged in studies of meritocratic ideology. Britain here emerges as the final destination of a postcolonial hierarchy where ‘Italy' was already a temporary destination, which lost appeal with the post‐2008 ′economic situation', when ‘many people' started emigrating.

This intergenerational perspective helps understand both meritocratic Britain's emotional power among participants, and why this cultural script includes apparently contradictory, more ‘structural' elements, like a search for better institutional support. The latter appears at odds with scholarship that stress the links between meritocracy and neoliberalism. However, this is not a contradiction if meritocracy is conceptualised as practical commonsense embedded in broader, ‘fuzzier' historical narratives, which include earlier discourses about equality of opportunity (Littler 2017). Moreover, practical knowledge is activated in relation to specific structural contexts, like experiences of class inequality. Awa, whose parents moved to Italy from Ivory Coast, remembered how her dad discussed Britain when the family moved there:

‘It's easier, the kids don't have to think about what to wear to school', it was kind of a joke, but he really liked it. He felt that here in England, they help their residents a bit more. There are benefits, they help children […] because in Italy, we used to go to school and pay for lunch, things like that, but in England sometimes it's free […] things like eating at school, even the uniform, which means you have your clothes and don't have to think about what you need to wear every day. These are small things but in the end they save costs.

The ‘England' evoked through the stories of Awa's father is associated with more sustained institutional support towards equality. ‘School uniforms' and welfare provisions (like free school meals) reduce economic and status inequalities; they are ‘small things' that can ‘save costs' and hence facilitate upward mobility. This narrative about support thus co‐exists with the idea, discussed above, that Britain also offers better educational and occupational opportunities (as reported also in recent research on the Italian‐Bangladeshis community in London; Morad et al. 2021).

Giovanni brings these two narratives together in his discussion of ‘meritocracy' in Britain. He uses the NHS as an example, where his parents had long‐term roles in housekeeping:

The NHS is quite a safe haven, both in terms of retirement and many other aspects. People find that there's a natural course of training and support, they've established a solid infrastructure […]. There are also other things that keep you in the workplace and help you take on certain roles, then it becomes a matter of choice, vocation and freedom, which aren't always present in Italy. In Italy, as an immigrant, you're already competing with Italians, which can be expected, but then obviously I can't say much about meritocracy there.

Like others, Giovanni stressed the visibility of ‘people of colour' in ‘important roles', but also elaborated on the ‘support' and ‘infrastructure' required to achieve ‘certain roles', combining a neoliberal understanding of merit ‐ centred on the prestige of middle‐class professions (Lamont et al., 2016) ‐ with social‐democratic understandings of security.

As discussed next, participants do not passively subscribe to these cultural scripts, but negotiate them vis‐à‐vis new contexts and life‐course transitions. However, the experience of being racialised as 'foreigners' in Italy emerged as central for understanding why meritocratic Britain remained an emotionally resonant narrative, despite new knowledge about Britain's inequalities.

## 7 Seeking Recognition: Double Consciousness, ‘Italian’ and ‘British’ Racism

Most participants experienced some educational and occupational mobility in Britain. While moving abroad in different circumstances, they mostly completed A levels or vocational qualifications and applied to UK universities, albeit largely in non‐elite institutions and almost always by taking on student debt. The majority also worked part‐time or full‐time during their studies (in hospitality, customer service or various forms of agency work), while some older participants eventually landed middle‐class jobs in areas like administration, marketing and teaching. Having parents with predominantly working‐class jobs (in Italy or later Britain), participants' trajectories took classed forms. Nevertheless, the story of meritocratic Britain maintained emotional resonance, even as they learned its contradictions via new information and social ties (Mijs and Usmani 2024). This section shows that the experience of *racialised self‐formation* in Italy and participants' search for recognition explain why learning about ‘British' racism did not challenge, per se, their emotional attachment to the script of meritocratic Britain.

Komal, whose parents moved to Italy from Pakistan, moved to Britain with her family when she was 15. She went through A‐levels, university and some work experience in corporate administration, and was now doing a PhD. Perhaps unsurprisingly, her views of Britain remained positive, given that the meritocracy story resonated with her achievements and trajectory. Yet, her views of British society were not one‐dimensional. When asked if Brexit had changed in any way her perceptions, she mentioned ‘the other side of the UK':

When we came, it was like, a really welcoming sort of feeling, right? I didn't know the other side of the UK […] there are people who are racist. There are people who are like, if there wasn't a law and if there wasn't this strict sort of idea that we belong to one community and community should be promoted and everything, I think it was dangerous place to live, very harsh people.

Komal explains how the early days' 'welcoming feeling' developed into a more nuanced view. Like others, she mentions 'the law' (Britain's equality legislation) as a protection against racial discrimination.^2^ However, she now sees legislation and the rhetorical promotion of ‘community' as more superficial aspects of British society, framing racism as something that underpins more polite interactions. She elaborated further on the actions she now takes, such as avoiding certain places, or thinking twice before applying for jobs in companies where 'they are all white people', or selecting her social circles:

Every community has that bubble, right? Food, you know, that street full of Pakistanis, full of Indians, full of like, Africans, you can stay in that bubble and be happy, you don't overthink about it, then you can still get the job, right? You're still less discriminated here than in Italy, I still believe that.

While Komal explains how racialised 'communities' act as a protection towards everyday racism, she makes clear she still believes you can achieve upward mobility (‘get the job') and be ‘less discriminated' than in Italy. New knowledge and changing social networks (Mijs and Usmani 2024) have qualified, but not challenged, Komal's view of Britain as more meritocratic. Alternatively, the literature on meritocratic ideology could interpret this discussion as a self‐interested defence of Komal's social mobility. However, Komal stressed that the experience of being treated as a ‘foreigner' in Italy remained central to her feelings about Britain. She introduced the issue by telling twice ‘I really need to say this to an Italian', thus acknowledging a ‘veil' (Du Bois, 1903/2007) that removes white Italians from the experience of growing up as 'foreigners' in Italy. Having still connections in Italy, she was concerned that what happened to her was now happening to her younger cousins:

[Italians] call them *stranieri* [foreigners], why do they use this word? This is so, so bad […] they came to me, they're like, we are *stranieri*, and I'm like why? […], you're Italian, you're born here, you're going to live your whole life here, you're gonna raise your kids here. Why are you calling yourself this? And this doesn't happen in the UK […] nobody calls me foreigner, ‘you're foreigner', they'll of course ask me what's your ethnicity. OK, you can ask that question, I'm from Pakistan, fine, but you don't [say] ‘you're a foreigner', so you don't belong here. That is the problem, I think it creates an inferiority complex, you know, maybe we are less of anyone. That's I think the point I really want to raise.

Other participants, particularly visible Muslim women like Komal and phenotypically Black participants, talked extensively about how, as children, they were addressed as 'foreigners', despite being born or growing up in Italy. Du Bois's double‐consciousness helps connect this foundational experience of misrecognition to socialisation processes. One learns from a young age that despite feeling Italian they are not recognised as such, which generates both what Komal calls ‘inferiority complex', and a ‘second sight' (Du Bois, 1903/2007) about the phenomenology of Italian racism, particularly how the repeated use of *straniero* reproduces status hierarchies between white and minoritised Italians.

Isaac evoked similarly ‘formative' experiences, despite being in a different position in Britain. While Komal had experienced middle‐class occupational contexts, Isaac was still working in hospitality and via agencies when we met. He moved to Britain alone about 10 years before, re‐enrolled into high school and went through university education in non‐elite universities (like others, taking on student debt). He had always lived in shared accommodation and, at the time of our conversation, was applying for his first graduate jobs. He acknowledged that things were getting ‘hard' in Britain, given rising prices and inflation. His emphasis on having to ‘work really hard' resonates with Ho's (2024) findings about meritocratic individualism as an emotional resource for working‐class youth, who craft ‘agentic selves' to navigate structural inequalities. However, while reflecting on the impact of cost of living and Brexit, Isaac stressed that compared to what he went through in Italy, these issues were ‘nothing':

It seems to me that things are always the same [in Britain] […] yes, now there's the crisis, but I don't know how, like, is affecting everyone I guess? I mean, yes, you feel the crisis, because obviously everything costs so much, rent, bills, the cost of living is hard, but as I said, compared to what I went through in Italy, what I'm going through now is nothing, it's nothing.

Isaac described the cost of living as ‘hard', but also as an equaliser (‘is affecting everyone'). While this view might obscure the unequal impact of economic crises, Isaac's comments should not be read in terms of accuracy of information, but as a story about (mis)recognition. This is why ‘compared to Italy' ‐ where he ‘was always attacked for my skin colour, always, from primary to high school' ‐ the things happening in Britain are ‘nothing'. Isaac also described more structural dimensions of Italian racism, like citizenship legislation, which prevents children of migrants from applying to Italian citizenship until they are 18. While Isaac eventually obtained Italian citizenship, he mentioned the experience of growing up having to regularly prove his and his family's status:

I don't know if people have told you, but for me the *questura* [police station] is hell, that place… I remember the day my mom got her permanent residence permit, there was a Nigerian guy who was yelling at the police officers […] they took someone else's photo and put it on his document, and when he confronted them, rightly pointing out that there was a mistake, they responded: ‘but you look all the same anyway' […] you have to be really strong, that's why I said that the things I went through in [hometown's name], in Italy, with people, friends, teachers, the society over there, once you come to England, England is nothing in comparison.

Like Komal, Isaac recognises a ‘veil' (Du Bois, 1903/2007) that removes white Italians from the experiences he is describing (‘I don't know if people have told you'). He highlights openly disrespectful forms of racism (‘you look all the same'), which are normalised by the *legal violence* of residence permits and citizenship applications (Menjívar and Abrego 2012). These stories are part of Isaac's memories about *becoming* Italian, hence they touch on an ontological sense of self (Du Bois, 1903/2007) that is unlikely to be challenged only by learning about ‘British' racism and inequalities.

Overall, participants went through experiences of *racialised self‐formation* in Italy that continued to inform their assessment of living in Britain. This pushed some participants towards a different practical narrative. ‘Racism is everywhere', as some remarked, but at least Britain offers a degree of invisibility through more ‘polite' interactions and formal respect via anti‐discrimination legislation. These aspects were seen as preferable to the openly disrespectful ‘Italian' racism and felt less painful than the experiences of racialised self‐formation described above.

Approaching these narratives about meritocratic Britain simply as false consciousness would obscure participants' experiences of self‐formation within structurally racist contexts. It would thus not capture the nuanced, comparative understanding of Britain and Italy that they developed through practical experience. Similarly, psychological interpretations of meritocracy as 'compensation' would decontextualise participants' legitimate claims to equality and security, thus trivialising them and obscuring their reflections on different types of European racism. The next section discusses the more complex mechanisms that challenge meritocratic Britain's emotional resonance.

## 8 Limits of Meritocratic Recognition: Leaving Britain

The participants who left Britain, or were thinking about it, mentioned a sense of cultural and social connectedness, associated with ‘home', that was not satisfied by the meritocratic promise (or reality) of more opportunities. Leaving Britain was thus triggered by feeling that occupational and educational mobility ‐ the practices historically associated with meritocratic recognition (Littler 2017) ‐ were not sufficient sources of meaning and identity.

Sara returned to Italy after finishing her degree because she progressively experienced a tension between ‘quality of life' and ‘job opportunities'. She recognised the intersubjective power of meritocratic Britain (‘everyone says come here, you'll make loads of money'). However, after her degree she realised that finding a job in her field was ‘very competitive' and that she might need to live in shared accommodation for a long time. Being ‘almost in [her] thirties', she felt these conditions were ‘not acceptable'.

Other aspects of ‘quality of life' convinced Sara to start sending job applications also to Italy. For instance, being aware that Italian migrants frequently dismiss differences in weather and food between Italy and Britain as 'complaints' from less resilient Italian migrants, she stressed that hers wasn't the ‘typical Italian' complain:

I like to explore new places, so I didn't act like the typical Italian who complains about parmesan, if you know what I mean, but looking at it in the long run […] the low quality of food and weather, which many people warned me about before I left, I thought, ‘how can the weather affect me? Can it really have an impact on your mood?' […] yes, it did, it made me very sad and I saw many people around me who were unwell, in terms of mental health. This is something that scared me because I thought there were different factors involved […] I saw a lot of people who were suffering, then I also thought, still with a future perspective, about the state of healthcare and how people were treated for even the smallest illnesses, let alone the bigger ones.

Sara evokes a different Britain than the meritocratic one: with a public health system that she perceived as less reliable than in Italy, and where several 'factors' negatively affect people's ‘mental health'. The practical experience of a colder weather also made her reconsider Italian narratives that dismiss this aspect of Britain as something that migrants supposedly overcome with self‐resilience. More broadly, she mentioned life‐course transitions (‘a future perspective') as important. As someone entering her thirties, she clarified: ‘when you are an adult, when you're thinking about having a family, obviously you need to consider these things'.

Sara eventually moved to Italy because she found a job that was in line with her degree and work experience. Work and social mobility hence remained important considerations, but they were negotiated via‐à‐vis other relational, cultural and temporal (future‐oriented) needs. Similarly, Gabriela returned to Italy one year after completing a degree in Britain. She pointed to similar ‘quality of life' issues, like: ‘not finding a sense of home, like feeling disoriented, especially after university, because you don't have the support networks you had in Italy'. Experiences of everyday racism (‘I was stopped multiple times in England […] in stores, compared to Italy') and growing knowledge of British political and Black popular culture further contributed to a revaluation of meritocratic Britain:

I worked for a year [after university] and I realised that, well, it wasn't a perfect society, and that discrimination still exists, even though… I like this quote that is often mentioned, from Black British rappers Stormzy and Dave, who say something like: ‘even if they say you are the least racist, you are still racist.' So even though England is seen by many Afro‐descendant Europeans as a key destination […] it still remains a racist place in the end, because clearly representation does not equal representativeness, and probably having a prime minister like Rishi Sunak explains 100% what the English model is all about: the divide and conquer of the British empire.

Only a minority of participants talked explicitly about British politics, and such participants had biographies of activism or social‐political science degrees. Hence, Gabriela gained access to political narratives about British racism and colonialism that were not readily available to most participants. This led her to include the ‘quality of life' issues within a wider critique of Britain's politics of ‘representation' and ‘divide and conquer' model, where a non‐white Prime Minister (Sunak) is acceptable as long as he does not challenge racial inequalities.

Overall, narratives about leaving Britain revealed the limits of meritocratic scripts of recognition: middle‐class jobs and feelings of progression through educational and occupational mobility did not fully capture a sense of connectedness, home and cultural comfort that some felt Britain was unable to offer, despite the experience of *racialised self‐formation* in Italy. Crucially, returnees were frequently individuals who left Britain at a key lifecourse transition (after finishing university) and who had moved abroad later than those who moved with their families as teenagers. This shows why the power of meritocratic ideology needs to be studied vis‐à‐vis temporal and biographical change, while recognising the tensions between meritocratic recognition and other sources of meaning and self‐worth.

## 9 Conclusion

This article argued that by rethinking meritocratic ideology as a form of practical, embodied knowledge, we can provide a less reductionist account of how people relate to narratives of (in)equality and under what conditions they acquire or lose emotional resonance. Rather than approaching meritocracy as a problem of (mis)information, false consciousness or psychological compensation, we argued that meritocratic commonsense appeals to legitimate desires for *recognition*, especially among groups that see it denied through histories of racial and class exclusion.

Drawing on Du Bois's double‐consciousness, this article showed that experiences of *racialised self‐formation* in Italy are central for understanding Black and Muslim Italians' 'second sight' into the workings of racism, and why they become invested in narratives of meritocratic Britain. These narratives revealed a composite postcolonial genesis: they are not exclusively neoliberal, but draw on post‐war discourses about equality and security in former colonial centres, which circulated within intergenerational histories of migration.

Meritocratic Britain emerged as a practical and emotional ‘map', tested through participants' tangible experiences of British society. Some participants developed nuanced accounts of the differences between 'Italian' and 'British' racism, yet still preferred to live in post‐Brexit Britain. Others missed the quality of life, relationships and sense of home associated with Italy and decided to return. Still others developed a political critique of Britain that complemented their concerns about quality of life. These paths were influenced by different life‐course stages and trajectories, making participants' relationship with meritocratic Britain contextual and temporally changing.

Taking advantage of its qualitative, biographical methodology, this article showed that meritocracy is not the only principle of recognition driving participants' considerations. Other sources of meaning and self‐worth, like sense of community and cultural comfort, also influenced their evaluations of social‐geographical mobility. By contrast, quantitative studies provide limited insight into the contexts of people's relationship with meritocracy, or how meritocracy interacts with other narratives of (in)equality (Zhu 2024). This risks overstating meritocracy's power and portraying social actors as overly rational and individualist, while confining their understandings of inequality to national borders.

More broadly, we suggest that by recovering Du Bois's understanding of misrecognition as producing double‐consciousness, rather than simply interiorised stigma, social scientists might move beyond ‘strong' notions of ideology and better understand how minoritised communities develop insights into inequality, adapting and transforming hegemonic narratives (like meritocracy) in their search for recognition. While Bourdieu helped us approach participants' stories as practical and temporally changing, Du Bois helped us recognise that symbolic violence is confronted through both narrative and concrete tactics: like packing one's bags and leaving (sometimes multiple times) in search of a better life.

Future research should explore further what narratives of (in)equality ‘do' in concrete circumstances and across the life‐course, while paying more attention to racialised minorities' experiences. This is especially important in colour‐blind European contexts that continue to ignore such experiences and their complexity, while reproducing racist exclusions.

## Funding

The first author received funding from the School of Social Sciences and Humanities, Loughborough University.

## Ethics Statement

This research was produced from a study which received ethical approval from a subcommittee of Loughborough University Ethics Committee. [Ethics reference: 2023‐15692‐15880].

## Conflicts of Interest

The authors declare no conflicts of interest.

## Permission to Reproduce Material From Other Sources

This authors have nothing to report.

## Data Availability

The data that support the findings of this study are available on request from the corresponding author. The data are not publicly available due to privacy or ethical restrictions.

## Endnotes

^1^
See Meer and Du Bois (2019) for further discussion of Du Bois's distinctiveness vis‐à‐vis mainstream conceptualisations of recognition (e.g., Fraser and Honneth 2003; Lamont and et al. 2016).

^2^
Participants with Eastern European backgrounds shared similar experiences but talked about them with partly different narrative repertoires, which are beyond this article's scope.

^3^
Black, Muslim and other minoritised Italians remain largely excluded from both temporary and permanent positions in Italian, European and UK universities. As white Italian academics based in Britain, we hope this small, largely self‐funded project contributes to challenging Italy's colour‐blindness and institutional racism, but also UK‐centric debates on racial inequalities that overlook the experiences of Black, Muslim and minoritised Europeans living in the UK.
